# The Foreign Journals: Chemistry

**Published:** 1839-04

**Authors:** 


### CHEMISTRY.

### *On the Pathological Alterations in the Constitution of Bones.*
By Professor SEBASTIAN, of Groningen.

THIS short paper contains the results of the chemical analysis of various specimens of bones, derived partly from man and partly from the lower animals; some of them being healthy and others diseased. It appears from them, that the relative proportions of the animal and earthy constituents of bone are altered by disease, and that this alteration consists in the augmentation of the proportion of the animal matter.

Professor Sebastian's experiments were conducted according to the dry method of analysis, and the results obtained were as follows:

| | Earthy matter. | Animal matter. |
|---|---|---|
| I. *Healthy bones of the lower animals.* | | |
| The long bones of the iguana contained . . | 60.00 | 40.00 |
| The ribs of the python . . . . | 50.00 | 50.00 |
| The shell of the land-tortoise . . . | 57.50 | 42.50 |
| The opercula of the haddock . . . | 60.00 | 40.00 |
| The furcula of a duck . . . . | 55.00 | 45.00 |
| The bone of the penis of a phoca . . . | 61.65 | 38.35 |
| The bone of the penis of a trichechus rosmarus . | 56.34 | 43.66 |
| The spinous process of a dolphin . . . | 60.00 | 40.00 |
| II *Healthy bones of man.* | | |
| The humerus contained . . . . | 63.34 | 36.66 |
| The thigh . . . . . | 63.34 | 36.66 |
| The tibia . . . . | 63.34 | 36.66 |
| The spongy substance of the same tibia , . | 66.66 | 33.34 |
| The bones of the skull (three experiments) . | 60.00 | 40.00 |
| The skull of an ancient Greek, excavated at Athens | 80.00 | 20.00 |
| III. *Diseased bones.* | | |
| A thigh-bone thickened by lues venerea contained . | 60.00 | 40.00 |
| A tibia ditto ditto . . | 73.24 | 26.66 |
| Another thigh-bone ditto ditto . . | 60.25 | 39.75 |
| A parietal bone ditto ditto . . | 60.93 | 39.07 |
| Another ditto ditto . . | 56.52 | 43.48 |
| A thigh-bone thickened in consequence of scrofula | 56.93 | 43.07 |
| An exfoliation of the tibia . . . | 60.87 | 39.13 |
| An anchylosed portion of the knee-joint . . | 56.25 | 43.75 |
| An anchylosed portion of the spinal column . | 60.00 | 40.00 |
| An exfoliated portion of the frontal bone . . | 60.00 | 40.00 |
| An exostosis of the skull . . . . | 62.88 | 37.12 |
| The callus which united the fractured neck of a thigh-bone | 41.66 | 58.34 |
| The callus of the os ilium of a woman . . | 65.39 | 34.61 |
| The upper fragment of a broken and badly-cured thigh-bone . . . . . | 57.82 | 42.18 |
| The bent tibia of a man . . . . | 62.00 | 38.00 |
| The macerated shoulder-blade of a dolphin . . | 56.66 | 43.34 |
| The ossified cartilages of the ribs of a horse . | 54.54 | 45.46 |

*Nat. Tijdschrift.* I. 4. 1838.

